# Transcriptome Sequencing and Characterization for the Sea Cucumber *Apostichopus japonicus* (Selenka, 1867)

**DOI:** 10.1371/journal.pone.0033311

**Published:** 2012-03-12

**Authors:** Huixia Du, Zhenmin Bao, Rui Hou, Shan Wang, Hailin Su, Jingjing Yan, Meilin Tian, Yan Li, Wen Wei, Wei Lu, Xiaoli Hu, Shi Wang, Jingjie Hu

**Affiliations:** 1 Key Laboratory of Marine Genetics and Breeding, College of Marine Life Sciences, Ocean University of China, Qingdao, China; 2 Beijing Genomics Institute at Shenzhen, Shenzhen, China; Temasek Life Sciences Laboratory, Singapore

## Abstract

**Background:**

Sea cucumbers are a special group of marine invertebrates. They occupy a taxonomic position that is believed to be important for understanding the origin and evolution of deuterostomes. Some of them such as *Apostichopus japonicus* represent commercially important aquaculture species in Asian countries. Many efforts have been devoted to increasing the number of expressed sequence tags (ESTs) for *A. japonicus*, but a comprehensive characterization of its transcriptome remains lacking. Here, we performed the large-scale transcriptome profiling and characterization by pyrosequencing diverse cDNA libraries from *A. japonicus*.

**Results:**

In total, 1,061,078 reads were obtained by 454 sequencing of eight cDNA libraries representing different developmental stages and adult tissues in *A. japonicus*. These reads were assembled into 29,666 isotigs, which were further clustered into 21,071 isogroups. Nearly 40% of the isogroups showed significant matches to known proteins based on sequence similarity. Gene ontology (GO) and KEGG pathway analyses recovered diverse biological functions and processes. Candidate genes that were potentially involved in aestivation were identified. Transcriptome comparison with the sea urchin *Strongylocentrotus purpuratus* revealed similar patterns of GO term representation. In addition, 4,882 putative orthologous genes were identified, of which 202 were not present in the non-echinoderm organisms. More than 700 simple sequence repeats (SSRs) and 54,000 single nucleotide polymorphisms (SNPs) were detected in the *A. japonicus* transcriptome.

**Conclusion:**

Pyrosequencing was proven to be efficient in rapidly identifying a large set of genes for the sea cucumber *A. japonicus*. Through the large-scale transcriptome sequencing as well as public EST data integration, we performed a comprehensive characterization of the *A. japonicus* transcriptome and identified candidate aestivation-related genes. A large number of potential genetic markers were also identified from the *A. japonicus* transcriptome. This transcriptome resource would lay an important foundation for future genetic or genomic studies on this species.

## Introduction

Echinoderms, which first appeared in the Early Cambrian [1] comprise about 7,000 living species and constitute the second-largest group of deuterostomes. They and their sister phylum, hemichordates are the closest known relatives of the chordates. Echinoderms are a special group of marine invertebrates. They occupy a taxonomic position that is believed to be important for understanding the origin and evolution of vertebrates. Much of our understanding of echinoderm genomics has come from the sea urchin *Strongylocentrotus purpuratus* (Echinodermata: Echinoidea). The *S. purpuratus* genome represents the first genome reported in a non-chordate deuteroztome, which has already provided new insights into some fundamental biological questions such as vertebrate evolution [2], [3], evolution of immune diversity [4], [5] and gene regulatory networks [6], [7]. However, to our knowledge, comparative genomics or transcriptomics among echinoderms remains lacking mostly due to limited genomic or transcriptomic resources for many species. Generating genomic or transcriptomic resources for a broader range of echinoderm species would clearly enable a better understanding of the phylogeny, speciation and diversification of deuterostomes.

Sea cucumbers are the representative echinoderms in the class Holothuroidea. There are approximately 1,250 species recorded worldwide with the highest number in the Asia Pacific realm. They play an important role in the ocean ecosystem by breaking down detritus and organic matter for bacteria and thus recycling nutrients back into the world's seas. Some sea cucumber species possess several interesting biological characteristics. For example, in response to high temperatures, some species can protect themselves by entering a physiological state called aestivation that is characterized by inactivity, feeding cessation, intestine degeneration, weight loss and metabolism rate depression [8]–[12]. In addition, some species possess another defensive mechanism called evisceration, that is, they can expel the internal organs (e.g., the intestine and respiratory trees) out of their body when they get stressed (e.g., chemical stress, physical manipulation, crowding) [9], [13], [14], [15], and the missing organs can be regenerated concurrently and rapidly afterwards [14]–[17]. In China, there are approximately 134 species of sea cucumbers with 28 of them considered edible or with medicinal properties [18].

The sea cucumber, *Apostichopus japonicus*, which is widely distributed along Asian coasts, has become one of the most important aquacultural species in China [19]. Genetic breeding programs, aiming to improve its growth rate and disease resistance, have just begun to be launched. Recent genetic studies have been focusing on the development of molecular markers [20], [21], construction of genetic maps [22], and characterization of immune-related genes [23], [24]. However, the progress of genetic studies on *A. japonicus* remains to be lagging in comparison with many other aquaculture species such as Atlantic salmon (*Salmo salar*), grass carp (*Ctenopharyngodon idella*) and Pacific oyster (*Crassostrea gigas*), partially due to the insufficient genetic or genomic resources. Fortunately, considerable efforts have recently been devoted to increasing the number of expressed sequence tags (ESTs) for *A. japonicus*. For example, Zheng et al. [13] sequenced 730 ESTs from two cDNA libraries to identify candidate genes that were potentially involved in intestine regeneration. Yang et al. [25] generated 5,728 ESTs from different tissues including intestine, body wall and respiratory tree, and identified 636 immune-related genes. However, the publicly available ESTs generated by the Sanger sequencing method (n = 7,716 in the GenBank database, as of 1/12/2012) are still insufficient to represent a whole transcriptome for *A. japonicus*.

As one of the next-generation sequencing (NGS) platforms, the Genome Sequencer FLX (GS FLX) System, powered by the 454 sequencing technology, has increased the sequencing throughput enormously and allowed collection of large amount of data rapidly and cost-effectively. Comparison with other NGS platforms such as Illumina's Solexa and ABI's SOLiD, 454 sequencing technology can produce much longer reads, and therefore is widely used for *de novo* transcriptome sequencing in non-model organisms [26]–[28]. Particularly, transcriptome profiling and characterization by 454 sequencing have been successfully applied in many marine species such as European eel *Anguilla anguilla* L. [29], Brown bryozoan *Bugula neritina* [30], Pandalid shrimp *Pandalus latirostris* [31], Manila clam R*uditapes philippinarum* [32] and *Yesso scallop* Patinopecten yessoensis [33].

Just recently, Sun et al. [15] performed the first large-scale transcriptome profiling for *A. japonicus* by 454 sequencing to identify candidate genes that were potentially involved in the intestine and body wall regeneration. Although more than 23,000 contigs were generated, their transcriptome data was unlikely to cover the whole transcriptome since only two tissues (i.e., intestine and body wall) were sequenced. Characterization of the whole transcriptome for *A. japonicus* would enable a full view of its transcriptome organization and provide the most complete gene sets to facilitate future genetic and genomic studies on this species. Obviously, accomplishment of this goal cannot be solely based on the publicly available EST data, and would require the transcriptome data derived from diverse sample origins. In this study, we performed a large-scale transcriptome sequencing of diverse *A. japonicus* cDNA libraries representing different developmental stages and adult tissues. After transcriptome assembly, 29,666 isotigs were obtained, and these isotigs were further clustered into 21,071 isogroups. Nearly forty percent of the isogroups were annotated with 6,963 unique gene names. In addition, 94% of the unique gene names were only appeared in our sequencing data, but not in the public EST data, indicating that our transcriptome data represents a more complete gene collection currently available for *A. japonicus*.

## Results and Discussion

### 454 Sequencing and assembling

**Figure 1:**
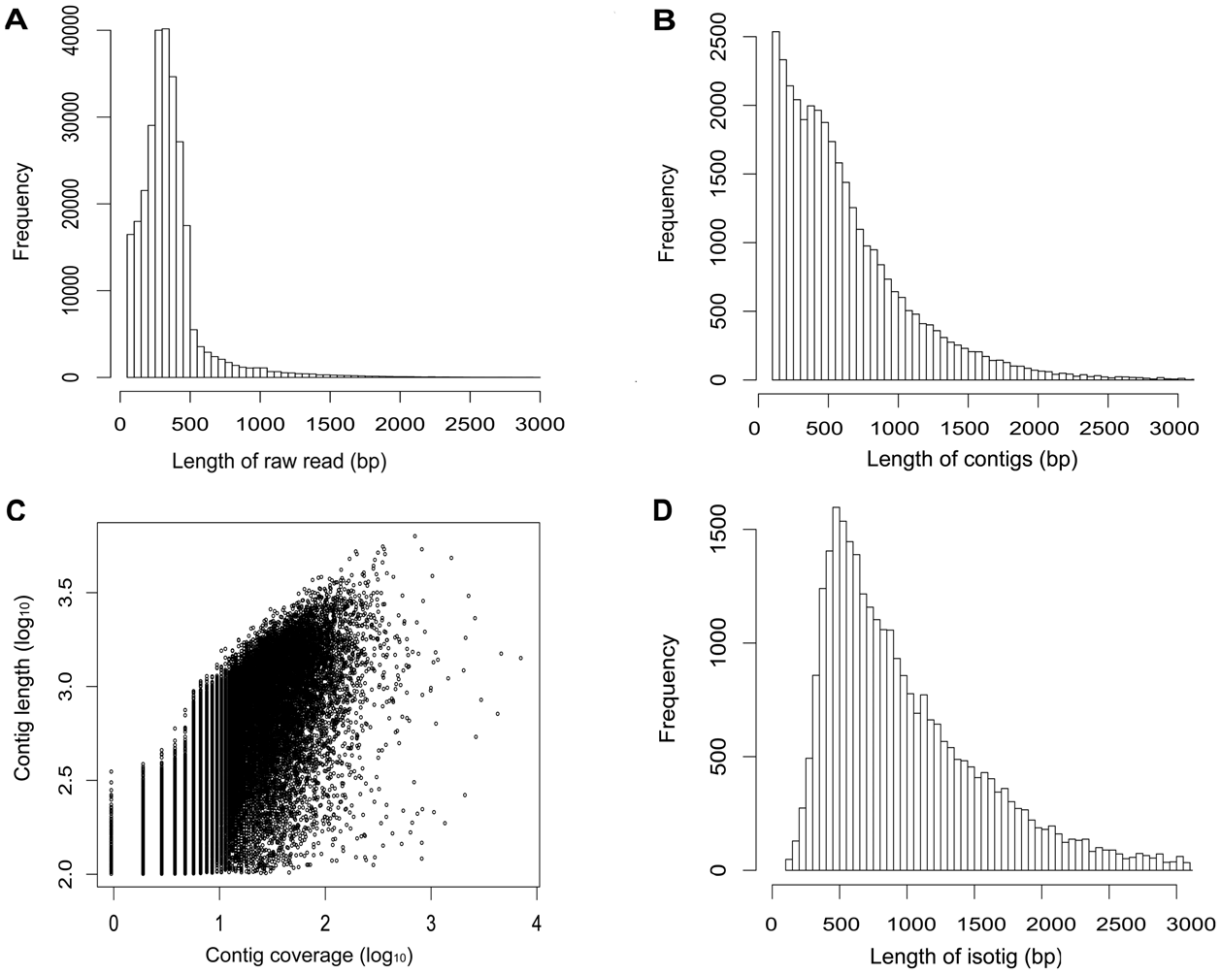
Overview of the *de novo* assembly of the *Apostichopus japonicus* transcriptome. (A) Size distribution of raw reads. (B) Size distribution of contigs. (C) Log-log plot showing the dependence of contig lengths on the number of reads assembled into each. (D) Size distribution of isotigs.

To maximize the transcript representation in a broad range of biological processes, eight *A. japonicus* cDNA libraries representing different developmental stages and adult tissues were constructed and used for 454 sequencing (Table 1). After a single-run of 454 sequencing, a total of 1,061,078 raw reads with an average length of 344 bases were obtained. The size distribution of raw reads is shown in Figure 1A. Of all these reads, 92% (974,004 reads) passed through our quality filters and represented high-quality (HQ) reads.

**Table 1:** Summary of eight *Apostichopus japonicus* cDNA libraries used for 454 sequencing.

Library	Developmental stages^a^/adult tissues	No. of individuals used for library construction	Normalization
L1	Embryo (4 h, 23 h)	∼100	Yes
L2	Larva (30 h, 6d, 8d, 10d)	∼100	Yes
L3	White juvenile (16d, 22d)	∼20	Yes
L4	Black juvenile (32d, 37d)	∼20	Yes
L5	Female gonads	7	Yes
L6	Male gonads	7	Yes
L7	Intestines, respiratory trees and coelomic fluid from active adults	6	No
L8	Intestines, respiratory trees and coelomic fluid from aestivating adults	6	No

a The sampling time after fertilization was indicated in the brackets for each developmental stage (h for hour and d for day).

The HQ reads combined with the public ESTs were assembled into 33,835 contigs and 199,011 singletons. Approximately 80% (774,993) of the HQ reads were incorporated into contigs. The size of contigs ranged from 101 to 6,323 bp, with an average length of 630 bp. The size distribution of contigs is shown in Figure 1B. The sequencing coverage of contigs ranged from 2 to 7,429 with a mean of 23. As expected for a randomly fragmented transcriptome, there was a positive relationship between the length of a given contig and the number of reads assembled into it (Figure 1C). Contigs were then assembled into 29,666 isotigs ranging from 104 to 7,697 bp with an average length of 1,042 bp (N50 = 1,294 bp). The size distribution of isotigs is shown in Figure 1D. The average contig coverage for each isotig was 2.1. The isotigs were further grouped into 21,071 isogroups, which possibly represents the total number of genes in the *A. japonicus* transcriptome. The summary statistics for the *A. japonicus* EST assembly are shown in Table 2.

**Table 2:** Summary statistics of transcriptome assembly for *Apostichopus japonicas*.

Category	Count
Assembled reads	974,004
Contigs	33,835
Contig size N50	844
Average length of contigs	630
Mean no. reads per contig	24.1
Singletons	199,011
Singleton size N50	332
Average length of singletons	297
Isotigs	29,666
Isotig size N50	1,249
Average length of isotigs	1,042
Mean no. contigs per isotig	2.1
Isogroups	21,071
Mean no. isotigs per isogroup	1.4

In comparison with the 454 sequencing data obtained from Sun et al. [15], 53% of the HQ reads obtained in our study did not find significant matches (Blastn, e<10^−4^) with their data. For gene annotation, 94% of gene annotations obtained in this study were not found in their data. These above results possibly suggest more representative collections of *A. japonicus* genes in this study.

### Gene annotation

Regarding gene annotation, all isogroups were searched against the Swiss-Prot database with an e-value threshold of 1e-6. Of 21,071 isogroups, 8,229 (39%) had at least one hit, which corresponded to 6,963 unigene names. The sequences and annotation information of all isogroups are provided in Dataset S1 and Table S1. More than half of the isogroups did not match to known genes most likely due to the fact that insufficient sequences are available in public databases from phylogenetically closely related species. The annotation rate in our study is also comparable to those (20∼40%) reported in the previous *de novo* transcriptome sequencing studies for non-model organisms [26], [27], [33], [34], [35].

**Figure 2:**
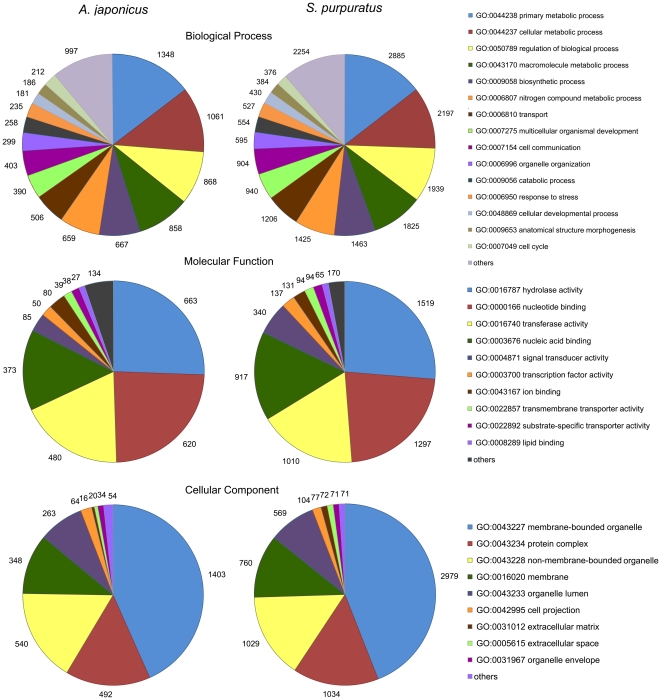
Gene ontology (GO) comparison (level 3) between the sea cucumber *Apostichopus japonicus* and the sea urchin *Strongylocentrotus purpuratus*. Transcriptome comparison revealed similar GO term representations between the two species.

Gene ontology (GO) annotation was further performed for the annotated isogroups in terms of biological process, molecular function and cellular component. In total, 4,167 isogroups were assigned with one or more GO terms for a total of 18,791 GO assignments. Distribution of the isogroups in different GO categories (level 3) is shown in Figure 2. For biological process, genes involved in the primary metabolic process and cellular metabolic process were highly represented. For molecular function, hydrolase activity was the most represented GO term, followed by nucleotide binding. Regarding cellular component, major categories were membrane-bounded organelle and protein complex. GO assignments for biological process suggested that 1% of the isogroups were assigned to the GO terms growth (GO: 0040007) and reproduction (GO: 0000003) (Table S2). These categories were of particular interest to researchers as growth and reproduction are important economic traits for sea cucumbers. Some important genes from these categories were found, such as myosin, major yolk protein, and Piwi-like protein 1. Interestingly, the major yolk protein, which usually plays a key role in egg development, was also detected in the male gonads, suggesting its potential role in the development of the *A. japonicus* male gonad.

Besides GO analysis, KEGG pathway analysis [36] was also carried out for the annotated isogroups, which is an alternative approach to categorize genes functions with the emphasis on biochemical pathways. Enzyme commission (EC) numbers were assigned to 2,146 isogroups, which were involved in 281 different pathways. The isogroups involved in these pathways is summarized in Table 3. Of these 2,146 isogroups with KEGG annotations, 37% were classified into the metabolic pathways with most of them involved in carbohydrate metabolism, energy metabolism, and amino acid metabolism. Genetic information processing including translation, folding, sorting and degradation, transcription, and replication and repair was represented by 35% of the KEGG annotated isogroups. Cellular processes were represented by 22% of the KEGG annotated isogroups. The transport and catabolism, cell growth and death, cell communication and cell motility were well-represented sub-pathways. In addition, 8% of the KEGG annotated isogroups were classified into immune system, which may be worthy of further investigation to search for disease-resistance genes in sea cucumbers.

**Table 3:** KEGG biochemical mappings for *Apostichopus japonicas.*

KEGG Pathways	Sub-pathways	Number of Unigenes	Number of isogruops
Metabolism	Carbohydrate Metabolism	136	170
	Energy Metabolism	128	145
	Amino Acid Metabolism	119	139
	Lipid Metabolism	117	163
	Nucleotide Metabolism	86	108
	Glycan Biosynthesis and Metabolism	82	106
	Metabolism of Cofactors and Vitamins	74	93
	Xenobiotics Biodegradation and Metabolism	45	67
	Metabolism of Other Amino Acids	42	58
	Metabolism of Terpenoids and Polyketides	14	16
	Biosynthesis of Other Secondary Metabolites	12	13
Genetic Information Processing	Translation	239	273
	Folding, Sorting and Degradation	238	264
	Transcription	123	139
	Replication and Repair	93	110
Cellular Processes	Transport and Catabolism	194	233
	Cell Growth and Death	108	140
	Cell Communication	104	128
	Cell Motility	52	68
Environmental Information Processing	Signal Transduction	205	250
	Signaling Molecules and Interaction	58	66
	Membrane Transport	16	18
Organismal Systems	Immune System	131	165
	Nervous System	114	146
	Endocrine System	111	137
	Digestive System	94	120
	Development	55	69
	Excretory System	55	69
	Circulatory System	48	62
	Sensory System	20	24
	Environmental Adaptation	19	20

### Differential expression analysis between the active and aestivating sea cucumbers

Aestivation is possibly a protection strategy for sea cucumbers to survive from high temperatures [10], [11]. Studies on sea cucumber aestivation from different perspectives such as morphology [10], [11], [37], physiology [10], [11], [37], [38], and immunology [39] have been performed. Nonetheless, the molecular mechanism of this process is still far from fully understood. Identification and characterization of candidate genes involved in aestivation would represent the first step to understand the genetic basis of aestivation in sea cucumbers.

Two non-normalized libraries (L7 and L8) were constructed in order to identify differential expressed genes between the active and aestivating sea cucumbers. The distribution of sequencing coverage for both libraries (Figure S1) revealed many genes considered to be lowly expressed. In order to ensure a reliable comparison, the isogroups with read counts less than 20 in both libraries were not included in the comparison, the validity of which was further justified by the Q-PCR validations. Transcriptome comparison revealed 446 differentially expressed genes (DGEs, FDR<0.05), of which 253 were down-regulated in aestivating sea cucumbers whereas 193 were up-regulated (Table S3). Approximately 64% of these genes (146 down-regulated and 141 up-regulated genes) have been annotated (Table S3). Nearly 50% of these genes were grouped into 33 GO categories (e.g., primary metabolic process, cellular metabolic process and regulation of biological process) representing diverse biological processes. The genes involved in stress response are of particular interest to us because these genes may function in protecting sea cucumbers from external stresses during aestivation. For example, superoxide dismutase (SOD) usually functions in removing excess superoxide anions produced during stress. Heat shock proteins (HSPs) (e.g., Heat shock protein beta-1) are responsible for protein folding, assembly, translocation and degradation in many normal cellular processes, stabilize proteins and membranes, and can assist in protein refolding under stress conditions [40]. There are also a number of un-annotated genes that are worthy of further investigation, as their expression level changed significantly when sea cucumbers went into aestivation.

**Figure 3:**
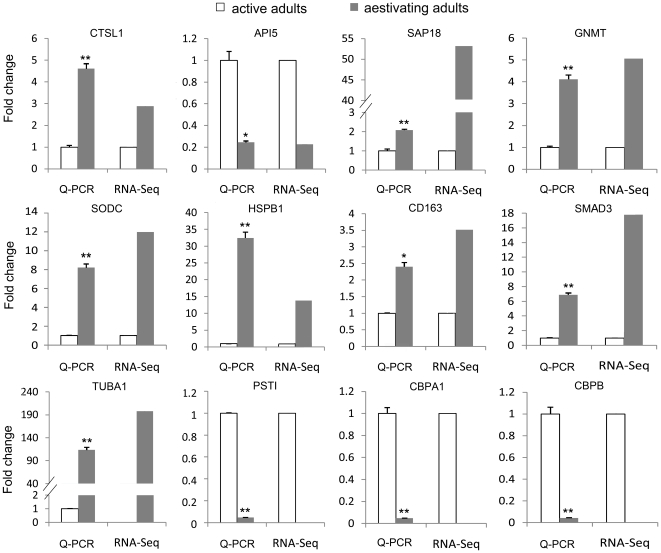
Quantitative real-time PCR (Q-PCR) validation of 12 genes that were differentially expressed between the active (white bar) and aestivating (grey bar) adult sea cucumbers. CTSL1, Cathepsin L1; API5, Apoptosis inhibitor 5; SAP18, Histone deacetylase complex subunit SAP18; GNMT, Glycine N-methyltransferase; SODC, Superoxide dismutase [Cu-Zn]; HSPB1, Heat shock protein beta-1; CD163, Scavenger receptor cysteine-rich type 1 protein M130; SMAD3, Mothers against decapentaplegic homolog 3; TUBA1, Tubulin alpha-1 chain; PSTI, aqualysin-1; CBPA1, Carboxypeptidase A1; CBPB, Carboxypeptidase B. For each Q-PCR validation, six technical replications were performed. Significant levels are indicated by * (*p*<0.05) and ** (*p*<0.01).

Twelve candidate DGEs were selected for Q-PCR validation. Q-PCR results verified the differential expression of these genes between the active and aestivating sea cucumbers (Figure 3). However, it should be noted that many genes in the two libraries were lowly expressed and therefore were not included in the comparison. More DGEs would be determined from these lowly expressed genes by performing deep transcriptome sequencing in future studies.

### Transcriptome comparison with the sea urchin Strongylocentrotus purpuratus

The sea urchin *Strongylocentrotus purpuratus* represents the only echinoderm species for which the whole genome sequence data is available [41]. To our knowledge, comparative genomics or transcriptomics has not yet been carried out among echinoderms mostly due to limited genomic or transcriptomic resources. Transcriptome comparison between the sea cucumber *A. japonicus* and the sea urchin *S. purpuratus* revealed similar transcriptome organization in terms of GO terms and their relative frequencies (Figure 2).

BlastX comparisons revealed that 42% (8,745) of the sea cucumber isogroups had significant matches to 29% (6,311) of the sea urchin proteins. Reciprocal tBlastN searches showed that 13,741 (63%) sea urchin proteins had significant matches to 5,685 (27%) sea cucumber isogroups. In total, 4,882 best matches were common in both comparisons, representing putative orthologous genes present in the two genomes. Additionally, 4% (202) of the putative orthologous genes identified in the two echinoderm species were not found in the non-echinoderm species, possibly representing the echinoderm-specific genes. However, as the *A. japonicus* isogroups may represent partial transcripts, the actual number of orthologous genes between the two species could be underestimated, which needs to be clarified by further studies.

### SSR and SNP detection

Transcriptome is also an important resource for rapid and cost-effective development of genetic markers. The molecular markers developed from the transcribed regions have the greatest potential for identifying functional genes that are responsible for economically important traits such as growth and reproduction.

A total of 730 SSRs were identified (Table 4). The largest fraction of SSRs identified was tri-nucleotides (44%), followed by tetra-nucleotide (22%). Among the SSRs identified, AC (9%) accounted for 42% of the di-nucleotide repeats, while ATC (24%), AAT (23%) and AAG (17%) accounted for 64% of the tri-nucleotide repeats. The most abundant other repeat motifs were ATAC (33%), AAAAC (8%) and AATCTC (14%), respectively. The average frequency of SSRs was found to be one SSR per 29.2 kb.

**Table 4:** Summary of SSRs identified from the *Apostichopus japonicus* transcriptome.

SSR Type	No. of SSR-containing ESTs	No. of SSRs	% of total SSRs
Dinucleotides	70	72	9.9
Trinucleotides	302	320	43.9
Tetranucleotides	144	157	21.5
Pentanucleotides	126	139	19.0
Hexanucleotides	35	42	5.7
Total	669	730	100

In addition, 52,102 high-quality SNPs and 2,083 indels were identified from 10,333 contigs (Table 5). The predicted SNPs included 30,366 transitions, 21,736 transversions. The overall frequency of all types of SNPs including indels was one per 393 bp. We randomly chose 32 contigs containing 201 SNPs to evaluate the preference of SNP position in the coding regions. The result showed that 70% of SNPs resided in the third codon position, while 18% and 12% in the first and second codon position, respectively. Experimental validation was also carried out to evaluate the reliability of the predicted SNPs. Fifteen of randomly chosen SNPs were subject to experimental validation in 48 *A. japonicus* adults using the high-resolution melting (HRM) genotyping method [42]. It turned out that 80% of these SNPs could be validated, suggesting the majority of our predicted SNPs are likely to be true SNPs.

**Table 5:** Summary of SNPs/Indels identified from the *Apostichopus japonicus* transcriptome.

type	Count	Frequency per kb
*Transition*		
C/T	15,249	0.72
A/G	15,117	0.71
*Transversion*		
A/T	7,444	0.35
A/C	5,452	0.26
T/G	5,367	0.26
C/G	3,473	0.16
*Indel*	2,083	0.01
Total	54,185	2.5

Among all the identified SSRs and SNPs, 135 (18%) SSRs and 18,199 (35%) SNPs were derived from annotated contigs. These SSRs and SNPs would be priority candidates for marker development.

In conclusion, a comprehensive collection of ESTs has been achieved for the sea cucumber *A. japonicus* using the 454 GS FLX platform, permitting gene discovery and characterization across a broad range of functional categories. In addition, our study identified a large number of SSRs and SNPs, from which genetic markers can be rapidly developed to serve as tools for further genetic or genomic studies on this species.

## Methods

### Ethics Statement

Not applicable. Our research did not involve human participants or samples.

### Sampling of sea cucumbers

In order to maximize the transcript representation for 454 sequencing, we collected a variety of *A. japonicus* samples representing different developmental stages and adult tissues (Table 1). The number of individuals used for library construction is listed in Table 1. To obtain samples representing different developmental stages, artificial fertilization and larval cultures were performed according to [43] using ∼100 sex-matured adults as breeding parents, which were provided by Xunshan Aquatic Product Group Co., Ltd. (Rongcheng, Shandong Province, China). Samples were collected at different developmental stages, including embryo, larva, white and black juveniles. Active and aestivating adults (115±20 g) were provided by Dr. Zunchun Zhou (Liaoning Ocean and Fisheries Science Research Institute, China), which were collected from Guanglu Island (Dalian, Shenyang Province, China) in July, 2009. Aestivating adults were characterized according to the observed phenotypes such as inactivity, feeding cessation, and intestine degeneration as described in previous studies [8], [10], [11]. The intestine, respiratory tree and coelomic fluid (0.5–1.0 ml per individual) collected from the active and aestivating adults represented the only available materials for library construction at the time we performed this study. All samples were flash frozen in liquid nitrogen and then stored at −80°C until analysis.

### Total RNA extraction, library construction and 454 GS-FlX sequencing

Total RNA was extracted from each sample using the TRIzol® Reagent (Invitrogen, CA, USA) by following the manufacturer's protocol. The quantity and quality of total RNA was measured using an Ultrospec™ 2100 *pro* UV/Visible Spectrophotometer (Amersham Biosciences, Uppsala, Sweden) and gel electrophoresis.

Eight 454 libraries (Table 1) were constructed by following the library preparation procedure as described in [27], [33]. Libraries L1∼L6 were normalized to prevent over-representation of the most common transcripts. Libraries L7 and L8 were not normalized in order to facilitate the identification of candidate transcripts that were potentially involved in aestivation. During library construction, a unique barcode was incorporated into each library for further discrimination of sequence originality. Approximately, 5 µg of the mixed libraries was sequenced using the Roche Genome Sequencer FLX system (Roche, Basel, Switzerland). All sequencing reads were deposited in the Short Read Archive (SRA) database (http://www.ncbi.nlm.nih.gov/sra/), which are retrievable under the accession number SRA046386.

### Sequence assembly and functional annotation

The raw 454 reads combined with public ESTs were first pre-processed by trimming adaptors and eliminating very short sequences (less than 100 bp).The pre-processed sequences were then subject to assembling using the program Newbler v2.5 (Roche) (cDNA assembly mode). Default assembly parameters were used with the minimum overlap length of 40 bp and the minimum sequence identity of 90%. The assembly program Newbler can account for alternative splicing by creating a hierarchical assembly composed of contigs, isotigs, and isogroups [44]. Contigs are stretches of assembled reads that are free of branching conflicts. An isotig represents a particular continuous path through a set of contigs (i.e., a transcript). An isogroup is the set of isotigs arising from the same set of contigs, (i.e., a gene). Different isotigs within an isogroup are considered to represent alternative isoforms of the same gene. All isotig sequences are provided in Dataset S1.

For gene annotation, to avoid redundant annotations, only one isotig (longest) from each isogroup was selected and compared against the Swiss-Prot database using BlastX with an E-value threshold of 1e-6. The top 20 hits extracted from the BlastX results were used for gene annotation and GO analysis (level 3) using the program Blast2GO [45], [46], which assigned GO terms describing biological process, molecular function and cellular component to the query sequences. In order to gain an overview of gene pathways networks, KEGG analysis was performed using the online KEGG Automatic Annotation Server (KAAS) (http://www.genome.jp/kegg/kaas/). The bi-directional best hit (BBH) method was used to obtain KEGG orthology assignments.

### Transcriptome comparison between active and aestivating sea cucumbers

In order to search for genes potentially involved in aestivation, relative expression abundance for each isogroup, which was calculated by dividing its read count by the total number of reads in a given library, was compared between the two libraries (L7 and L8) derived from active and aestivating adults, respectively. Isogroups with read counts less than 20 in both libraries were excluded from the comparison. Differential expression analysis was conducted using a web tool IDEG6 (http://telethon.bio.unipd.it/bioinfo/IDEG6_form/) [47] with the Audic and Claverie test [48] selected. To account for multiple testing, false discovery rate (FDR) was calculated using the QVALUE program [49].

Quantitative real-time PCR (Q-PCR) validation was performed for 12 selected candidate DGEs. Primers were designed using the Primer5 software (Premier Biosoft International), and all primer sequences have been listed in Table S4. Q-PCR reactions were performed in a 20 µl volume composed of 4 ng of cDNA, 4 µM of each primer, and 1× SYBR Green Master mix (TaKaRa) in the ABI 7500 Real time PCR System. Melting curve analysis was performed by the end of each PCR to confirm the PCR specificity. Six technical replications were performed for each Q-PCR validation. The *cytochrome b* (*cytb*) gene was used as the reference gene according to [23], [24]. The relative expression of target genes was calculated using the 2-DDCt method [50]. Differential expression between groups was determined using the one-tailed T-test (*p*<0.05).

### Transcriptome comparison with the sea urchin Strongylocentrotus purpuratus

The sea urchin *S. purpuratus* represents the only echinoderm species for which the whole genome sequence is available [41]. The protein sequences of *S. purpuratus* (v2.1 genome assembly) were downloaded from the NCBI FTP site. GO annotation of the *S. purpuratus* protein sequences were performed using the program Blast2GO [45], [46]. For transcriptome comparison, a reciprocal comparison approach [27] was adopted to identify putative orthologous genes. Briefly, the *A. japonicus* isogroup sequences were first compared with the *S. purpuratus* protein sequences using BlastX. Then a similar comparison of the *S. purpuratus* protein sequences against the *A. japonicus* isogroup sequences was performed using tBlastN. Pairs of putative orthologous genes were identified based on the reciprocal best matches with an e-value threshold of 1e-6. To search for genes that are present in both echinoderms, but not in the non-echinoderm species, the sea urchin proteins that extracted from the orthologous gene pairs were compared against the Swiss-Prot database using BlastP with the e-value threshold of 1e-4.

### SSR and SNP detection

All types of SSRs from dinucleotides to hexanucleotides were detected from the contigs using the program SciRoko [51] with default settings (for all repeat types, minimum total length = 15 bp and minimum repeats = 3). Potential SNPs were detected using the program GS Reference Mapper (v 2.6) with default parameters (cDNA mode). The SNP identification was limited to the contigs containing at least eight reads for each allele and required the minor allele frequency ≥25%. Fifteen predicted SNPs were randomly selected for experimental validation using a cost-effective high-resolution melting (HRM) genotyping method [42]. Primers and probes were designed using the Primer5 software (Premier Biosoft International), and all primer and probe sequences have been listed in Table S5. SNP validations were performed in 48 *A. japonicus* adults collected from Qingdao (Shandong Province, China). Genomic DNA extraction and HRM genotyping was conducted by following the protocols described in [20] and [42], respectively.

## Supporting Information

Dataset S1 **Assembled isotig sequences.**
(ZIP)

Figure S1 **Distribution of isogroup coverage for the active (blue) and aestivating (red) adults libraries.**
(TIF)

Table S1 **Gene annotation information obtained by comparing the isogroups against the Swiss-Prot database.**
(XLS)

Table S2 **Candidate genes involved in growth and reproduction.**
(XLS)

Table S3 **Differential expressed genes identified by transcriptome comparison between the active and aestivating sea cucumbers.**
(XLS)

Table S4 **PCR primers used for Q-PCR validation.**
(PDF)

Table S5 **PCR primers and probes used for validation of the predicted SNPs.**
(PDF)
