# Supplementary figures and images for: Transcriptome Sequencing and Characterization for the Sea Cucumber Apostichopus japonicus (Selenka, 1867)
Source: PLoS One. 2012 Mar 12;7(3):e33311. doi: 10.1371/journal.pone.0033311 (PMC3299772; doi:10.1371/journal.pone.0033311)

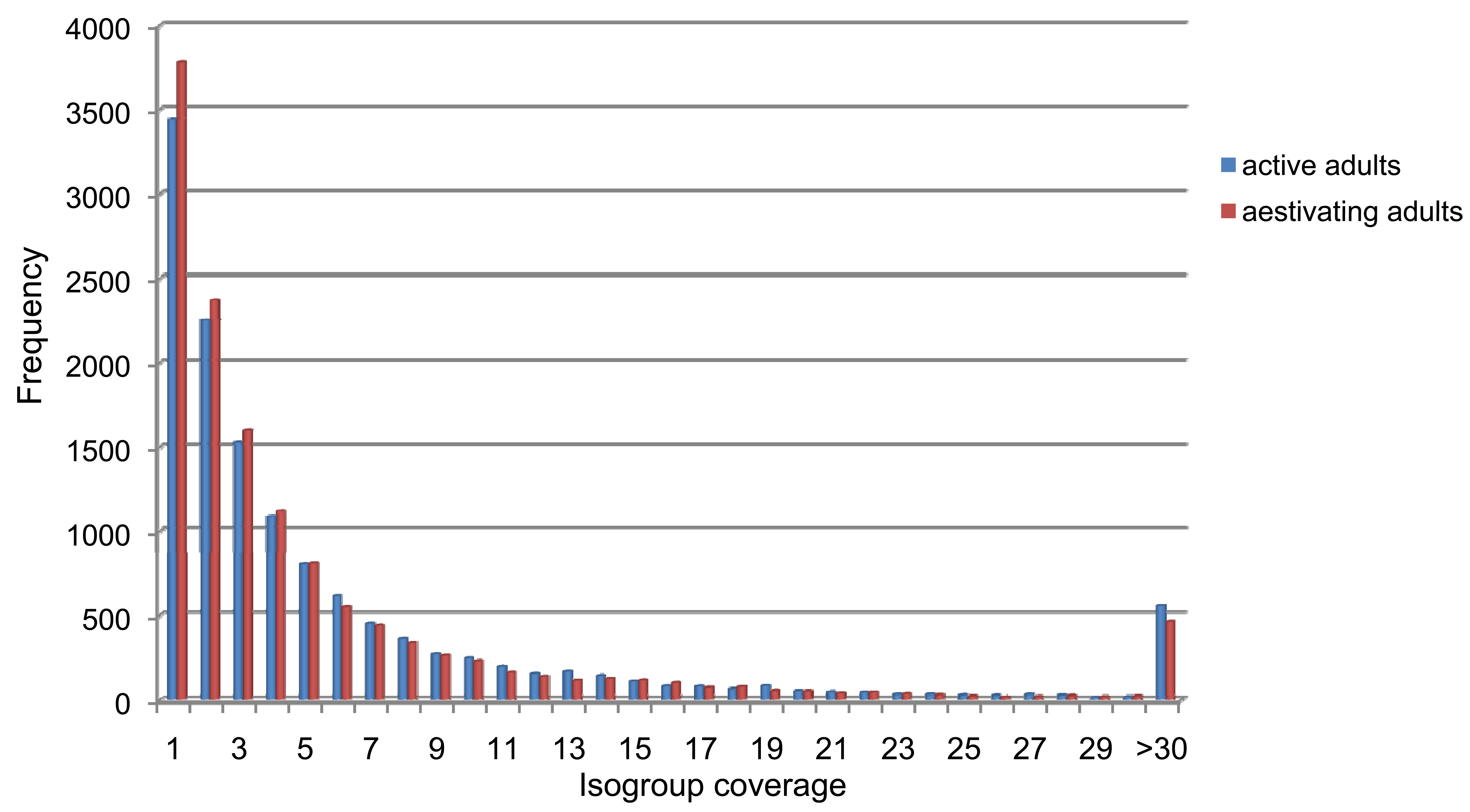

Supplement: Figure S1 — Distribution of isogroup coverage for the active (blue) and aestivating (red) adults libraries. (TIF) [file pone.0033311.s002.tif]
