# Supplementary material for: Transcriptome Sequencing and Characterization for the Sea Cucumber Apostichopus japonicus (Selenka, 1867)
Source: PLoS One. 2012 Mar 12;7(3):e33311. doi: 10.1371/journal.pone.0033311 (PMC3299772; doi:10.1371/journal.pone.0033311)
Supplement: Table S4 — PCR primers used for Q-PCR validation. (PDF) [file pone.0033311.s006.pdf]

Table S4. PCR primers used for Q-PCR validation.

Genes	Primer sequences	Product size (bp)
Cathepsin L1 (CTSL1)	F: 5’-GAGCCAGTAATCCTTCCCGTC-3’<br>R: 5’- GCTGCCCTCAGTTCGTTCC-3’	135
Apoptosis inhibitor 5 (API5)	F: 5’-CAGGAACAAAGGACGAGGTCG-3’<br>R: 5’- CACGGATATTCCACAAATTCGC-3’	178
Histone deacetylase complex subunit SAP18 (SAP18)	F: 5’-TGAGAAGACTAGAGGCATGGGAC-3’<br>R: 5’- GCATTGAGTAAATCAGAATCTGCC-3’	162
Glycine N-methyltransferase (GNMT)	F: 5’-CGGCAGTAGGAAGAGCAGAAC-3’<br>R: 5’- CATCTTATCCGAGGCGTCAAT-3’	169
Superoxide dismutase [Cu-Zn] (SODC)	F: 5’-ACTACACGGCTTTCACATCCAC-3’<br>R: 5’- CCATTATCATCGGCTTCCACA-3’	156
Heat shock protein beta-1 (HSPB1)	F: 5’-CCTGATTCTTCTTCCATCGCTTC-3’<br>R: 5’- TAACGTCGAGGCCCCAAGAC-3’	174
Scavenger receptor cysteine-rich type 1 protein M130 (CD163)	F: 5’-GTCAGTGGACCGTGAGTATGGC-3’<br>R: 5’- CGTCCCTGATCCTGGTTGG-3’	139
Mothers against decapentaplegic homolog 3 (SMAD3)	F: 5’-CCATCCAAAGCGTCTGTTGC-3’<br>R: 5’- AGCAGGGACCTTTCCATTGAG-3’	147
Tubulin alpha-1 chain (TUBA1)	F: 5’-ATTTGGTTGGCTGGCTCG-3’<br>R: 5’- TCCATTACTGCATCCTTGCG-3’	197
aqualysin-1 (PSTI)	F: 5’-GGTTTTCCGCCACCTATCGT-3’<br>R: 5’- AGCAAGTCAGGCAGCCCTAA-3’	149
Carboxypeptidase A1 (CBPA1)	F: 5’-TGTGAAGCCCTGAAGAAAGTGG-3’<br>R: 5’- CAAATCCGTAGTCTCCCGTGTC-3’	172
Carboxypeptidase B (CBPB)	F: 5’-CGAATGTGGAGAAAGACCCG-3’<br>R: 5’- TGCAGAAGTGCCCATGTAAGTG-3’	144
**Cytochrome b[a] (CYTb)**	F: 5’-ACCACACACCCGAATAGAGACC-3’<br>R: 5’- TTCGTAGCGGTCCTGTCATTT-3’	147

[a] The *CYTb* gene was used as the reference gene in the Q-PCR validation.
