# Supplementary material for: Transcriptome Sequencing and Characterization for the Sea Cucumber Apostichopus japonicus (Selenka, 1867)
Source: PLoS One. 2012 Mar 12;7(3):e33311. doi: 10.1371/journal.pone.0033311 (PMC3299772; doi:10.1371/journal.pone.0033311)
Supplement: Table S5 — PCR primers and probes used for validation of the predicted SNPs. (PDF) [file pone.0033311.s007.pdf]

Table S5. PCR primers and probes used for validation of the predicted SNPs.

SNP-ID[a]	Major/minor allele	Primer and probe sequences
C03388S227	T/C	F: 5’-TGCAGTGATGAGGAAGATGC-3’
		R: 5’-TTTTCGATGGGGAGTCAAAG-3’
		Probe: 5’-ACCATGGGAGGACAGGTTCCACAGAGA-3’
C04043S108	A/G	F: 5’-GAGGAGGATGGTAGTGGT-3’
		R: 5’-GTACAATATCTACATTGTCA -3’
		Probe: 5’-AGATTCAGATTGGATATCCTCCTTCTCGA-3’
C04043S419	A/T	F: 5’-CTCGACATTCGATGGCAAC-3’
		R: 5’-GAAGAGGCGTTGGCACTTAG-3’
		Probe: 5’-CTCCTGGAAGAATCTGTTAAAACAGTCCTAG-3’
C04044S77	T/A	F: 5’-TCTCCTCTACCCGTCCAA-3’
		R: 5’-TATATACCAACTGGAACTTG -3’
		Probe: 5’-CCTCATATTATTCCTGTTCTTTGTGTTCAATA-3’
C05139S49	A/G	F: 5’-AAATCCCTCTGCGCACAAT-3’
		R: 5’-AGTGCCAGCGCACTTATTTT-3’
		Probe: 5’-AGGCAGCCAATGACCTGACCATCGA-3’
C05139S108	A/G	F: 5’-CTGACCATCCTGTATTAACTTAACG-3’
		R: 5’-GGGGATGGACTTCCAAACT-3’
		Probe: 5’-GCACTGATCTGCCCTGATTGGTTGAC-3’
C06306S336	T/C	F: 5’-CTGTTGCCGATGAAACAATAG-3’
		R: 5’-GCAAATTGAACGAGATGGAG-3’
		Probe: 5’-CAGAATATCCTGCCTTGGGATAACGTAAACGATC-3’
C07952S361	T/G	F: 5’-GACTGATCTGGTAAACGAACGA-3’
		R: 5’-CACTACCTGGGAGGACGATT-3’
		Probe: 5’-CCGGGATACTCGGGGAGATTCAGGA-3’
C08347S758	G/C	F: 5’-ATTGAGCTCCATCCACTGCT-3’
		R: 5’-GCCGATCTGGTGAACCTAGT-3’
		Probe: 5’-CAGGAAGACATGCTGCAATTTTGCATC-3’
C10704S274	T/C	F: 5’-CCTCTCCATCTCCTCTGTCACT-3’
		R: 5’-GTAAAGGTGGCTGCCAGATG-3’
		Probe: 5’-TGACACTCTCTGACTCCAACCTCTGTCC-3’
C13728S402	A/C	F: 5’-CAGGTCAACCAGAAATATTACACA-3’
		R: 5’-TGTCGTCAGTTTTGATAAGCA-3’
		Probe: 5’-ACATAAGGCAGTGCCGTTAAACCTTAAACC-3’
C14781S689	C/T	F: 5’-CTAGCAGGATGAACAGAAT-3’
		R: 5’-AGTCTAGTTCACTGTGCTCCA -3’
		Probe:5’-TTGAGGAATCATCTTCATATGCCAATCATTT-3’

[a] SNP markers are named as follows: C followed by several numbers refers to a contig number, and then S followed by several numbers refers to the SNP position (bp) in this contig.
